# Diagnostic yield of an insertable cardiac monitor in a large patient population

**DOI:** 10.1016/j.hroo.2022.11.005

**Published:** 2022-11-24

**Authors:** Dennis H. Lau, Bertrand Pierre, Pilar Cabanas, Eimo Martens, Giovanni Bisignani, Daniel Hofer, Antonio Berruezo, Romain Eschalier, Jacques Mansourati, Thomas Gaspar, Victor Manuel Sanfins, Andrejs Erglis, Andreas Hain, Georgios Papaioannou, Alessandro Cuneo, Verena Tscholl, Jürgen Schrader, Thomas Deneke, BIO|MASTER.BIOMONITOR III study and BIO|STREAM.ICM registry investigators

**Affiliations:** ∗ Department of Cardiology, Royal Adelaide Hospital and the University of Adelaide, Adelaide, Australia; † Department of Cardiology, Tours University Hospital, Chambray-lès-Tours, France; ‡ Department of Cardiology, Hospital Álvaro Cunqueiro, Vigo, Spain; § Klinikum rechts der Isar, Technical University Munich, Munich, Germany; ¶ Department of Cardiology, Ospedale Civile Ferrari, Castrovillari, Italy; ‖ Department of Cardiology, University Hospital Zurich, Zurich, Switzerland; ∗∗ Department of Cardiology, Teknon Heart Institute, Barcelona, Spain; †† Department of Cardiology, Hôpital Gabriel Montpied, Clermont-Ferrand, France; ‡‡ Department of Cardiology, University Hospital of Brest, Brest, France; §§ Heart Center, University of Dresden, Dresden, Germany; ¶¶ Department of Cardiology, Hospital Senhora da Oliveira – Guimarães, Guimarães, Portugal; ‖‖ Department of Cardiology, Pauls Stradins Clinical University Hospital, Riga, Latvia; ∗∗∗ Department of Cardiology, Kerckhoff-Klinik GmbH, Bad Nauheim, Germany; ††† Department of Cardiology, Hôpital Saint-André, Bordeaux, France; ‡‡‡ Department of Cardiology, Krankenhaus Maria-Hilf Stadtlohn, Stadtlohn, Germany; §§§ Department of Cardiology, Charité Universitaetsmedizin, Campus Mitte, Berlin, Germany; ¶¶¶ Department of Cardiology, Biotronik SE & Co.KG, Berlin, Germany; ‖‖‖ Department of Cardiology, Rhön Clinic Campus Bad Neustadt, Saale, Germany

**Keywords:** Insertable cardiac monitor, Implantable loop recorder, Remote monitoring, Home monitoring, Cardiac arrhythmia

## Abstract

**Background:**

Insertable cardiac monitors (ICMs) are increasingly used for cardiac rhythm diagnosis with expanding indications. Little has been reported about their use and efficacy.

**Objective:**

The study sought to evaluate the clinical utility of a novel ICM (Biotronik BIOMONITOR III) including the time to diagnosis in unselected patients with different ICM indications.

**Methods:**

Patients from 2 prospective clinical studies were included to determine the diagnostic yield of the ICM. The primary endpoint was time to clinical diagnosis per implant indication or to the first change in atrial fibrillation (AF) therapy.

**Results:**

A total of 632 patients were included with a mean follow-up of 233 ± 168 days. Of 384 patients with (pre)syncope, 34.2% had a diagnosis at 1 year. The most frequent therapy was permanent pacemaker implantation. Of 133 patients with cryptogenic stroke, 16.6% had an AF diagnosis at 1 year, resulting in oral anticoagulation. Of 49 patients with an indication for AF monitoring, 41.0% had a relevant change in AF therapy based on ICM data at 1 year. Of 66 patients with other indications, 35.4% received a rhythm diagnosis at 1 year. Moreover, 6.5% of the cohort had additional diagnoses: 26 of 384 patients with syncope, 8 of 133 patients with cryptogenic stroke, and 7 of 49 patients with AF monitoring.

**Conclusion:**

In a large unselected patient population with heterogeneous ICM indications, the primary endpoint of rhythm diagnosis was achieved in ∼1 in 4, and additional clinically relevant findings was achieved in 6.5% of patients at short-term follow-up.

## Graphical abstract

**Figure:**
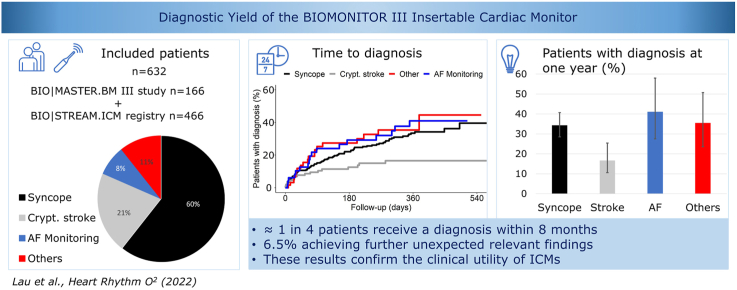

**Key Findings**

▪ The diagnostic yield of an insertable cardiac monitor in a large population with heterogeneous indications was ∼1 in 4 within 8 months.
▪ Additional incidental diagnoses were achieved from the insertable cardiac monitor in 6.5% of the patients.
▪ The overall diagnostic yield of an insertable cardiac monitor ranged from 16.5% to 41% at 12 months.

## Introduction

The use of insertable cardiac monitors (ICMs) is increasing due to expanding clinical indications from unexplained syncope to detection of subclinical atrial fibrillation (AF) in cryptogenic stroke, management of AF, and clarification of suspected symptomatic arrhythmias. The available literature on ICMs is focused on specific clinical settings or according to implant indications, but the diagnostic yield of ICMs in populations with heterogenous implant indications is not well reported.^1^, ^2^, ^3^, ^4^, ^5^ The BIOMONITOR III (Biotronik, Berlin, Germany) is a novel ICM with a miniaturized shape and a simplified insertion procedure.^6^^,^^7^ Here, we evaluate the clinical efficacy of this ICM regarding the time to diagnosis in 632 unselected patients with different ICM indications and compare the diagnostic yield for atypical indications with that for the most common indication of unexplained syncope.

## Methods

### Patient selection

The present analysis includes patients with a BIOMONITOR III device inserted as part of the completed BIO|MASTER.BIOMONITOR III study (NCT04025710) or the ongoing Observation of clinical routine care for patients with BIOTRONIK implantable cardiac monitors (BIO|STREAM.ICM) registry (NCT04075084). Both studies were initiated to satisfy postmarket clinical follow-up requirements for regulatory purposes and their enrollment criteria were minimal, including any clinical indication for an ICM implant, ≥18 years of age, and patient acceptance of remote monitoring. The subject must not be pregnant or be participant of another clinical study. To be included in the present analysis, patients had to have signed informed consent, and to have the insertion of the device plus any follow-up data reported by May 6, 2022. The research was conducted according to the principles of the Declaration of Helsinki with Institutional Ethics Committee approval at each site.

### Biomonitor III

The main characteristics of the BIOMONITOR III ICM are a large sensing vector (∼78 mm, composed of a 47.5-mm-long rigid component and a 30.5-mm-long flexible antenna adaptable to the curvature of the body), a miniaturized ICM profile (8.3 mm × 4.3 mm, weight 5 g), and a dedicated tool for a simplified injection-like insertion procedure with pocket formation and ICM placement in 1 step.^6^^,^^7^ The performance of this device from the implant procedure to sensing performance and patient acceptance has been reported recently in 653 individuals.^7^ The device has built-in algorithms to detect 5 different types of heart rhythm disturbances: bradycardia, pause, AF, high ventricular rate, and sudden ventricular rate drop.^6^^,^^7^ A 1-minute electrocardiogram (ECG) strip is recorded upon arrhythmia detection, and 7.5-minute ECG recordings can be triggered by the patient. The ICM uses the established Biotronik Home Monitoring system to automatically transmit up to 6 ECG strips, arrhythmia detection statistics, and sensing performance parameters once a day via wireless links without active patient participation.^8^

### Data collection and evaluation

In both the BIO|MASTER.BIOMONITOR III study and BIO|STREAM.ICM registry, the activation of the Home Monitoring feature was mandatory. The BIO|MASTER.BIOMONITOR III study enrolled patients between October 2019 and February 2021, with follow-up visits planned for the first month and at 3 and 12 months. The last patient completed the study on April 6, 2022, and the current publication was initiated when the data of the BIO|MASTER.BIOMONITOR III study were complete. The BIO|STREAM.ICM registry has been enrolling patients since October 2019 and will continue for a longer period. It collects long-term real-life data without mandatory study procedures and follow-ups; a patient completes the study when the investigator decides to explant the ICM or not to use ICM information any further.

For the present analysis, ICM indications were classified as (1) syncope or presyncope (hereinafter referred to as syncope), (2) screening for AF after cryptogenic stroke, (3) monitoring and management of AF, and (4) others. Investigators reported when the respective diagnosis had been made based on a detection of the ICM (for example, pause, bradycardia, or tachyarrhythmia detection in syncopal patients, or AF detection followed by oral anticoagulation therapy prescription in cryptogenic stroke). Specifically, the classification or diagnostic criteria for all arrhythmias and pauses were left to the individual investigator’s discretion. In patients with other indications, a diagnosis was individually compared with the indication, for example, AF detection in patients with palpitations. The primary endpoint of the present analyses was the time to the clinical diagnosis based on ICM information. Only in patients with an ICM for AF management, the primary endpoint was the first relevant change in AF therapy including change of antiarrhythmic medication, direct current cardioversion, or catheter ablation procedures.

### Statistical analysis

Data were used as entered until May 6, 2022. For the time-to-event analyses, patients were censored at the last study contact (eg, in-office or remote follow-up, diagnosis, adverse event, study termination). Other results are presented with standard summary statistics (mean ± SD, median [interquartile range] according to data distribution) and as absolute and relative frequencies. In the time-to-event analyses, 6-month and 1-year probability estimates according to Kaplan-Meier are provided with 95% confidence intervals. The analyses were performed using the R statistical software (version 2022.07.2, R Foundation for Statistical Computing, Vienna, Austria).

## Results

### Patients

This study included 632 patients (166 from the BIO|MASTER.BIOMONITOR III study, 466 from the BIO|STREAM.ICM registry) from 55 investigational sites in 11 countries, predominantly Germany (25.8%), France (17.0%), Spain (8.3%), and Australia (7.3%). The patients were 64 ± 16 years of age and 42.4% were women (Table 1). The ICM indications were syncope (60.8% of patients), cryptogenic stroke (21.0%), management of AF (7.8%), and others (10.4%). The indications summarized as others (n = 66) included palpitations (n = 20), clarification of ventricular tachyarrhythmia (VT) (n = 12) or supraventricular tachyarrhythmia (SVT) (n = 17), search for AF after peripheral embolism (n = 5), possible arrhythmias (n = 3), clarification of bradycardia or conduction system disturbances (n = 2), and miscellaneous (n = 7). Patient characteristics differed considerably across the 4 indication groups, with those with ICM implanted for AF monitoring having more cardiovascular risk factors such as hypertension, heart failure, diabetes, valvular heart disease, and coronary artery disease.

**Table 1:** Baseline characteristics of included patients according to ICM indication

	All Patients (N = 632)	(Pre)syncope (n = 384)	Cryptogenic stroke (n = 133)	AF monitoring and management (n = 49)	Other (n = 66)
Age, y	63.9 ± 16.3	65.1 ± 17.3	62.3 ± 14.3	64.6 ± 12.1	59.3 ± 16.4
Female	268 (42.4)	169 (44.0)	48 (36.1)	21 (42.9)	30 (45.5)
BMI, kg/m^2^	27.6 ± 5.4	27.2 ± 5.2	27.5 ± 4.9	29.7 ± 6.2	28.6 ± 6.4
Hypertension	367 (58.2)	215 (56.1)	80 (60.2)	36 (73.5)	36 (54.5)
CAD	119 (18.9)	78 (20.3)	13 (9.8)	20 (40.8)	8 (12.1)
Heart failure	84 (13.3)	51 (13.3)	9 (6.8)	15 (30.6)	9 (13.6)
NYHA functional class I/II/III/IV	18/37/13/2	13/20/7/1	2/5/1/0	1/8/3/1	2/4/2/0
Valvular disease	97 (15.3)	62 (16.1)	10 (7.5)	18 (36.7)	7 (10.6)
Stroke or TIA	176 (27.8)	31 (8.1)	133 (100)	6 (12.2)	6 (9.1)
COPD	33 (5.2)	23 (6.0)	6 (4.5)	1 (2.0)	3 (4.5)
Diabetes	105 (16.6)	61 (15.9)	23 (17.3)	13 (26.5)	8 (12.1)
History of arrhythmia					
AF	128 (20.3)	74 (19.3)	4 (3.0)	49 (100)	1 (1.5)
Other SVT	89 (14.1)	50 (13.0)	11 (8.3)	10 (20.4)	18 (27.3)
VT	51 (8.1)	28 (7.3)	1 (0.8)	3 (6.1)	19 (28.8)
Sick sinus disease	15 (2.4)	12 (3.1)	0 (0.0)	2 (4.1)	1 (1.5)
Any conduction system disturbance	160 (25.3)	115 (29.9)	13 (9.8)	14 (28.6)	18 (27.3)

Values are mean ± SD, n (%), or n.

AF = atrial fibrillation; BMI = body mass index; CAD = coronary artery disease; COPD = chronic obstructive pulmonary disease; ICM = insertable cardiac monitor; NYHA = New York Heart Association; SVT = supraventricular tachyarrhythmia; TIA = transient ischemic attack; VT = ventricular tachyarrhythmia.

### Diagnoses

At the time of data freeze, the 632 patients had a mean follow-up of 233 ± 168 days (median 220 [interquartile range 76-365] days). Of all patients, 583 had an indication other than AF monitoring, and 135 (23.2%) of them received an ICM-based diagnosis for the clinical question for which the device was implanted. Table 2 shows the number of patients for each indication and the diagnostic yield.

**Table 2:** Diagnostic yield in 632 patients with follow-up

ICM indication	Patients with diagnosis or change in therapy	6-mo estimates (%)∗	12-mo estimates (%)∗
(Pre)syncope (n = 384)	98 (25.5)†	22.1 (17.9–27.2)	34.2 (28.5–40.7)
Cryptogenic stroke (n = 133)	18 (13.5)†	12.6 (7.7–20.1)	16.6 (10.6–25.4)
AF monitoring (n = 49)	19 (38.8)‡	29.2 (18.0–45.1)	41.0 (27.5–58.0)
Other (n = 66)	19 (28.8)†	27.4 (17.4–41.5)	35.4 (23.6–50.7)

Values are n (%), unless otherwise indicated.

AF = atrial fibrillation; ICM = insertable cardiac monitor.

∗ Kaplan-Meier estimates of the proportion of patients with a diagnosis with 95% confidence interval.

† Diagnosis.

‡ Change in therapy.

**Figure 1:**
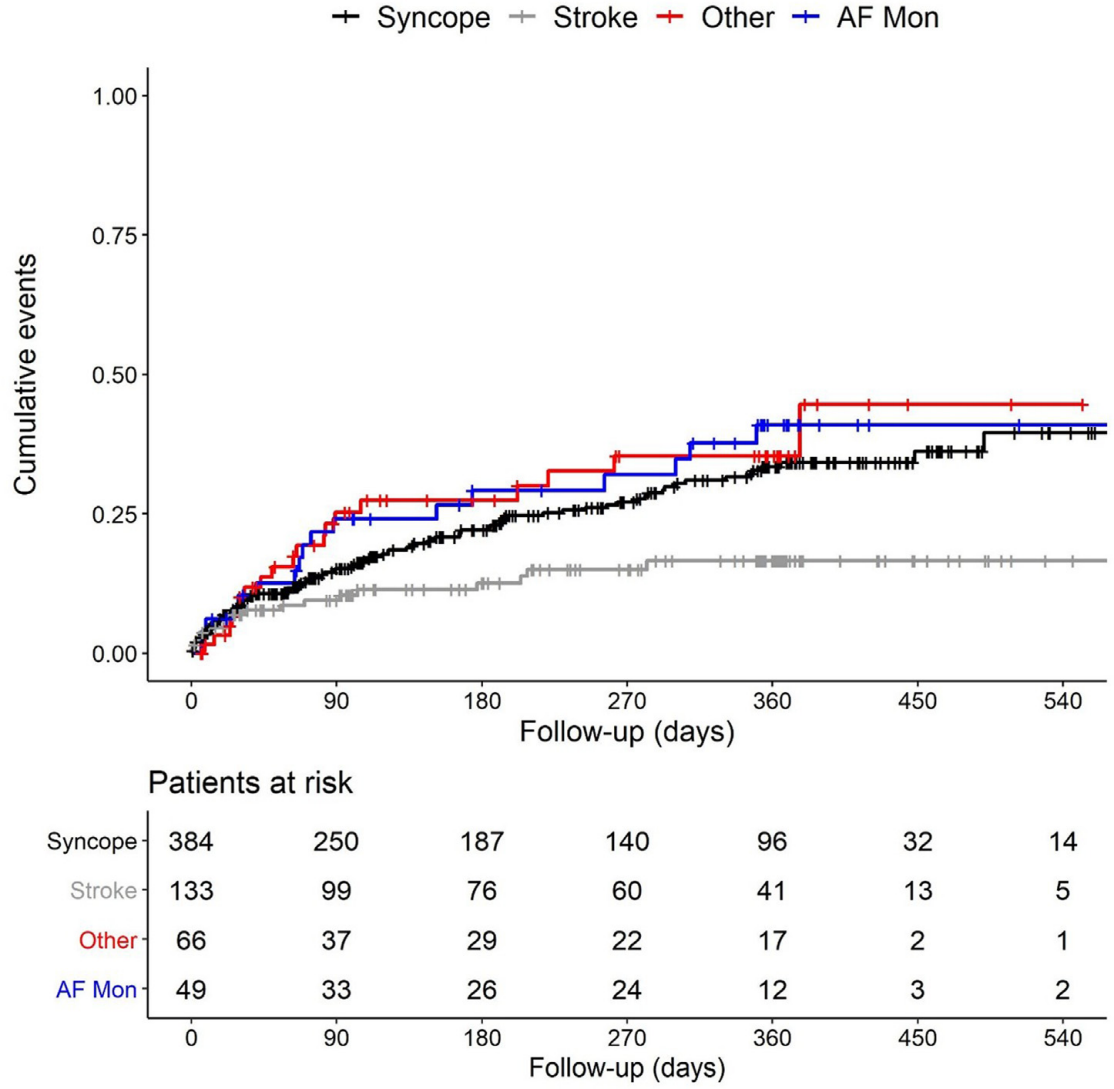
Kaplan-Meier plots of the time to diagnosis for the 3 insertable cardiac monitor indication groups: syncope or presyncope (red), cryptogenic stroke (green), and other indications (yellow). The fourth plot (blue) is the time to the first relevant change in atrial fibrillation (AF) therapy in patients who received an insertable cardiac monitor for monitoring and management of known AF.

More specifically, of 384 patients with syncope indication, 98 (25.5%) received a diagnosis based on the ICM. The ICM-detected arrhythmias were pause (n = 69 [70.4%]), bradycardia (n = 18 [18.4%]), SVT other than AF (n = 5 [5.2%]), VT (n = 3 [3.1%]), AF (n = 1 [1.0%]), and other or not specified (n = 2 [2.0%]). As a result, reported medical interventions included permanent pacemaker implantation (n = 71), medication change (n = 12), catheter ablation (n = 3), and cardioverter-defibrillator implantation (n = 3). The Kaplan-Meier estimates of the proportion of patients with a diagnosis were 22.1% at 6 months and 34.2% at 1 year (Table 2, Figure 1). In 7 patients, a syncope was reported without documentation of an arrhythmia by the ICM. Notably, 26 of these patients (6.8% of 383) had AF detected and received treatment accordingly.

Of 133 patients with cryptogenic stroke, 18 (13.5%) had an AF diagnosis based on the ICM. In all cases, oral anticoagulation was subsequently prescribed, and 2 patients also underwent catheter ablation for AF. The Kaplan-Meier estimates of the diagnostic yield were 12.6% at 6 months and 16.6% at 1 year (Table 2). In addition, other non-AF diagnoses from the ICM in 8 (6.0%) of 133 patients with cryptogenic stroke resulted in the following interventions: (1) closure of patent foramen ovale following exclusion of AF after 1 year of ICM monitoring in 2 patients; (2) permanent pacemaker implantation following detection of a significant pause in 4 patients; (3) coronary artery bypass grafting in 1 patient following detection of VT and subsequent coronary angiography; and (4) detection of a regular SVT with ventricular rate of 200 beats/min in 1 patient.

Of 49 patients with an indication for AF monitoring, 19 (38.8%) had a relevant change in AF therapy based on ICM data. Ten patients underwent 11 catheter ablation procedures for AF (n = 7), atrial flutter (n = 2), atrioventricular nodal re-entry tachycardia (n = 1), and frequent premature ventricular complexes (n = 1). Six patients underwent AF cardioversions, and 1 patient was started on a new antiarrhythmic medication. The Kaplan-Meier estimates of the diagnostic yield were 29.2% at 6 months and 41.0% at and 1 year (Table 2), respectively. Further ICM-based diagnoses in 7 (14.2%) of 49 patients with AF monitoring indication resulted in implantation of a permanent pacemaker (n = 6) or a cardioverter-defibrillator (n = 1).

Of 66 patients with other indications, 19 (28.8%) received a diagnosis based on the ICM. The diagnoses were AF (n = 6), SVT (n = 4), pause (n = 3), VT (n = 2), bradycardia (n = 1), sinus tachycardia (n = 1), ventricular extrasystoles (n = 1), and absence of VT recurrence following ablation (n = 1). The Kaplan-Meier estimates of the diagnostic yield were 27.4% at 6 months and 35.4% at 1 year. Concerning different types of indication, 6 of 26 patients with palpitations had heterogeneous arrhythmias diagnosed (2 AF, 1 SVT, 1 VT, 1 pause, 1 ventricular extrasystoles) and 3 of 7 patients after peripheral embolism had an arrhythmia diagnosis (2 AF, 1 SVT).

## Discussion

In our study of 632 patients with heterogeneous ICM implant indications over an average of 233 days of follow-up, we obtained the following principal findings. First, the primary endpoint achieved per implant indication was ∼1 in 4 with 25.6% for syncope, 13.5% for AF in cryptogenic stroke, 38.8% for relevant change in AF management based on AF monitoring, and 28.8% for the remaining indications. Second, additional incidental ICM diagnoses were achieved in 6.5% of the cohort: 26 (6.8%) of 384 patients with syncope, 8 of 133 patients with cryptogenic stroke (6.0%), and 7 (14.3%) of 49 patients with AF monitoring. Third, the overall diagnostic yield of ICM per implant indication ranged from 16.6% to 41.0% at 12 months. Taken together, cardiac rhythm monitoring with ICM appears to contribute to successful diagnosis and resultant management changes that are likely to improve patient outcomes.

The most established indication for ICM use is syncope, whereby prolonged monitoring is more likely to yield an arrhythmic diagnosis than intermittent monitoring. In our population, most patients (∼61%) received the device for the clarification of the cause of a syncope or presyncope. In a large meta-analysis of 41 studies of adults who underwent ICM implantation for unexplained syncope, the proportion of subjects finally diagnosed with arrhythmic syncope was 26.5%, ranging from 5.4% to 55.6%.^2^ Our results confirm good diagnostic yield for this ICM indication, with 22.0% of patients having an arrhythmic diagnosis at 6 months and 34.2% (95% confidence interval 27.8%–40.0%) of patients having an arrhythmic diagnosis at 1 year.

The second-largest group comprised patients with cryptogenic stroke (∼21%). AF detection in our cohort was 12.6% at 6 months and 16.6% (95% confidence interval 10.6%–25.4%) at 1 year. These numbers are slightly higher than those of the landmark Cryptogenic Stroke and Underlying AF (CRYSTAL AF) study (8.9% at 6 months, 12.4% at 1 year)^9^ and match the findings of a subsequent large observational study (12.2% at 6 months, 16.3% at 1 year).^10^ In our study, all patients had oral anticoagulation prescribed after AF diagnosis, to prevent further thromboembolic event.

A relatively small group of patients (∼8%) received the device for the monitoring of known AF, as this indication is not reimbursed in some of the participating countries. This is a relatively new application of ICM, typically in patients after AF ablation, in whom clarification of AF burden can influence disease managment.^1^^,^^4^^,^^5^^,^^11^^,^^12^ Nearly 1 in 5 patients underwent a repeat ablation during the relatively short follow-up period in our study, suggesting that the ICM can have a relevant influence on the management of these patients. Interestingly, there was a relatively high yield of cardiac conduction abnormalities or ventricular arrhythmia in this cohort, resulting in implantation of cardiac implantable electronic devices. This is not surprising, as these conditions often share common risk factors that lead to atrial remodeling.

However, the most interesting results in our study may be those pertaining to the remaining patients. The reason why these patients received their device were symptoms other than syncope that could be a consequence of unknown arrhythmias, or a physician’s interest to describe more precisely known or assumed arrhythmias (to improve diagnosis). In a few cases, the proof of AF was attempted in patients that did not have a cryptogenic stroke but had peripheral embolism. What patients in this heterogeneous indication group had in common was that their symptomatic burden and clinical risks were judged by physicians to be high enough to justify the insertion of an ICM. The ICM contributed to diagnosis in 27.4% of these patients at 6 months and 35.4% at 1 year, which is even numerically slightly higher than the diagnostic yield in patients with syncope.

Importantly, a considerable number of patients in our study received an unexpected diagnosis. The ICM provides added benefits due to the embedded algorithms to detect various rhythm-related abnormalities. This result affirms a role for not disabling the built-in algorithms beyond the main indication for the ICM implantation, although this decision may result in an increased ECG classification workload with likely additional device alerts.^13^

There are concerns that an invasive procedure should not be easily justified for a purely diagnostic device. The timescale of the time to diagnosis in our cohort makes clear that extracorporeal devices cannot be worn long enough to yield the same diagnoses. However, the low complication rate of approximately 1 serious adverse device effect in 100 patients reported from a previous analysis of our population (which were mostly device extrusions due to insufficient wound closure) is reassuring^7^ and should be weighed against a diagnosis rate of approximately 1 in 4 patients, as demonstrated in this analysis.

### Study limitations

Some limitations must be noted. First, we combined data from 2 studies with a different design. Further, some patients contribute only a relatively short follow-up duration, as the BIO|STREAM.ICM registry is ongoing. Nevertheless, the large number of patients is a strength, and the knowledge of the diagnostic yield and resultant outcome of the patients are clinically relevant.

### Conclusion

This study confirms the clinical utility of ICMs in a large population of patients with heterogeneous indications with ∼1 in 4 resulting in relevant diagnosis per implant indication and additional 6.5% achieving additional clinically relevant findings at short-term follow-up.

## References

[1] Ciconte G., Giacopelli D., Pappone C. (2017). The role of implantable cardiac monitors in atrial fibrillation management. J Atr Fibrillation.

[2] Solbiati M., Casazza G., Dipaola F. (2017). The diagnostic yield of implantable loop recorders in unexplained syncope: a systematic review and meta-analysis. Int J Cardiol.

[3] Giancaterino S., Lupercio F., Nishimura M., Hsu J.C. (2018). Current and future use of insertable cardiac monitors. J Am Coll Cardiol EP.

[4] Bisignani A., De Bonis S., Mancuso L., Ceravolo G., Bisignani G. (2019). Implantable loop recorder in clinical practice. J Arrhythm.

[5] Sakhi R., Theuns D.A.M.J., Szili-Torok T., Yap S.C. (2019). Insertable cardiac monitors: current indications and devices. Expert Rev Med Devices.

[6] Mariani J.A., Weerasooriya R., van den Brink O. (2020). miniaturized implantable cardiac monitor with a long sensing vector (BIOMONITOR III): insertion procedure assessment, sensing performance, and home monitoring transmission success. J Electrocardiol.

[7] Deneke T., Cabanas P., Hofer D. (2022). New-generation miniaturized insertable cardiac monitor with a long sensing vector: insertion procedure, sensing performance, and home monitoring transmission success in a real-world population. Heart Rhythm O2.

[8] Sogaard P., Behrens S., Konyi A. (2019). Transmission and loss of ECG snapshots: remote monitoring in implantable cardiac monitors. J Electrocardiol.

[9] Sanna T., Diener H.C., Passman R.S. (2014). Cryptogenic stroke and underlying atrial fibrillation. N Engl J Med.

[10] Ziegler P.D., Rogers J.D., Ferreira S.W. (2017). Long-term detection of atrial fibrillation with insertable cardiac monitors in a real-world cryptogenic stroke population. Int J Cardiol.

[11] Wechselberger S., Piorkowski C., Pohl M. (2016). Current rare indications and future directions for implantable loop recorders. Herzschrittmacherther Elektrophysiol.

[12] Assaf A., Theuns D.A.M.J., Sakhi R. (2022). Accuracy of atrial fibrillation detection by an insertable cardiac monitor in patients undergoing catheter ablation: results of the BioVAD study. Ann Noninvasive Electrocardiol.

[13] O'Shea C., Middeldorp M.E., Hendriks J.M. (2021). Remote monitoring alert burden: an analysis of transmission in >26,000 patients. J Am Coll Cardiol EP.

